# Choosing Between Tricuspid Repair or Replacement: Decision Algorithms

**DOI:** 10.1016/j.shj.2025.100732

**Published:** 2025-09-30

**Authors:** Hannah Kempton, Lukas Stolz, Ludwug Weckbach, Thomas Stocker, Philipp Doldi, Michael Näbauer, Steffen Massberg, Fabien Praz, Jörg Hausleiter

**Affiliations:** a Medizinische Klinik und Poliklinik I, LMU Klinikum, LMU München, Munich, Germany; b German Center for Cardiovascular Research (DZHK), Partner Site Munich Heart Alliance, Munich, Germany; c Department of Cardiology, Bern University Hospital, Bern, Switzerland

**Keywords:** Tricuspid regurgitation, Transcatheter tricuspid valve intervention, Transcatheter tricuspid edge-to-edge

## Abstract

Transcatheter tricuspid valve (TV) interventions have transformed the treatment of TV disease. Among available therapies, transcatheter tricuspid edge-to-edge repair has shown favorable safety, symptom relief, quality-of-life improvement, and reduction of heart failure hospitalizations in selected patients with severe tricuspid regurgitation (TR). In complex cases, repair may not result in optimal TR reduction, whereas transcatheter TV replacement (TTVR) offers TR elimination, irrespective of the valve anatomy. However, TTVR is less widely available, requires long-term anticoagulation, and can lead to periprocedural adverse events. As device options expand, careful procedural selection has become increasingly important and must be guided by a comprehensive, multidisciplinary assessment. Key factors include TV anatomy, right ventricular function, pulmonary pressures and resistance, right ventricular–pulmonary artery coupling, endocardial device leads, bleeding risk, imaging quality, and anticoagulation tolerance. Multimodal imaging, including echocardiography and cardiac computed tomography, along with right heart catheterization is an essential step for procedural planning. In addition, preprocedural optimization with diuresis, rhythm control, and collaboration with heart failure and electrophysiology specialists are essential to ensure optimal procedural outcomes. Transcatheter tricuspid edge-to-edge repair and TTVR should be viewed as complementary therapies that each play a role in tailoring transcatheter TV intervention to individual anatomical and clinical patient characteristics. This review presents a framework based on evidence and experience for procedural selection, highlighting the importance of individualized evaluation and multidisciplinary care. A stepwise algorithm is proposed to support decision-making in patients with severe TR.

## Highlights

• Transcatheter tricuspid edge-to-edge repair is both safe and effective in the majority of cases.
• Transcatheter tricuspid valve replacement gives more complete tricuspid regurgitation reduction, and expands options if transcatheter tricuspid edge-to-edge repair is not feasible.
• Device choice is individualized by anatomy, hemodynamics, and clinical risk profile.
• Anticoagulation and diuretic strategy are critical for management after transcatheter tricuspid valve replacement.

## Introduction

Moderate or greater tricuspid regurgitation (TR) occurs in approximately 4% of patients over the age of 75 years^1^, ^2^, ^3^, ^4^ and is associated with morbidities and mortality, contributing to a high burden of heart failure (HF) symptoms and hospitalizations.^5^ Surgical intervention for isolated TR has historically been underutilized, in part due to high operative risk, with a procedural mortality of 10%-20%, and therefore a perception of limited benefit.^6^^,^^7^ Recent advances in transcatheter technologies have changed the therapeutic landscape for TR.

Transcatheter tricuspid edge-to-edge repair (T-TEER) is well-established as a safe and effective treatment option, demonstrating improvements in symptoms, functional status, quality of life, and HF hospitalizations in patients with symptomatic severe or greater TR.^8^, ^9^, ^10^ Nevertheless, T-TEER has anatomical limitations such as large coaptation gaps, leaflet tethering, or severe annular dilation. To address these challenges, transcatheter tricuspid valve (TV) replacement (TTVR) devices have been developed as an alternative strategy that provides complete TR reduction, irrespective of the valve anatomy.^11^ However, TTVR is less widely available, requires long-term anticoagulation, and can lead to periprocedural adverse events. As the spectrum of transcatheter therapies expands, it is increasingly clear that a single strategy cannot be applied to all patients, and procedural and device selection must be guided by careful assessment of individual anatomical, technical, and clinical aspects. In this review, we examine the current evidence supporting T-TEER and TTVR, outline their respective indications and limitations, and propose a framework for selecting the optimal transcatheter approach for patients with symptomatic severe TR.

## Comparison of Procedural Outcomes

T-TEER is a well-established treatment modality for patients with symptomatic, severe, or greater TR.^8^, ^9^, ^10^ The TRILUMINATE pivotal clinical trial demonstrated an improvement in HF symptoms and quality of life for patients with severe TR treated with TriClip (Abbott, Chicago, IL).^9^^,^^12^ Procedural success, defined as TR reduction to moderate or less, was achieved in 88.9% of patients, and TR was reduced to mild or less in approximately 50% of patients. Although there were no consistent improvements in hard clinical endpoints at 1 year,^9^ the 2-year data showed a significantly lower annualized rate of HF hospitalizations in the T-TEER cohort.^13^

Similar results were reported in the PASTE registry using the PASCAL system with procedural success in 87% of patients, which was sustained in 83% at 1 year. Similarly, the Tri.FR trial, comparing T-TEER with TriClip to optimal medical therapy (OMT), reported procedural success in 78.6%, which was similarly sustained in 78.3% at 1 year.^14^

By comparison, the TRISCEND-II trial, as the pivotal trial for the first commercially available TTVR system (EVOQUE valve: Edwards Lifesciences, Irvine, CA), showed that TR was reduced to mild or less in 95.2% of TTVR patients. This was associated with a greater improvement in functional status and quality of life, consistent with findings from T-TEER trials that quality of life improvement is dependent on the degree of TR reduction after transcatheter TV intervention (TTVI).^15^, ^16^, ^17^, ^18^ Longer-term follow-up data are awaited to determine whether there is an impact on HF hospitalization or mortality that was not observed at 1 year.

### Mortality

The 30-day mortality rate for T-TEER in the TRILUMINATE trial was 1.1%, with an all-cause mortality of 9.4% at 1 year.^9^^,^^12^ In the large, prospective bRIGHT registry, which enrolled 511 patients with advanced comorbidities and predominantly massive or torrential TR, the 1-year all-cause mortality was 15.1%. Notably, patients who achieved moderate or less TR at 30 days had significantly lower mortality at 1 year, emphasizing the prognostic importance of successful TR reduction. The bRIGHT registry also demonstrated that T-TEER was associated with substantial improvements in functional status and quality of life at 1 year, even in a real-world, high-risk population.^19^ The EuroTR registry stratified patients into 3 disease stages, using the criteria that were externally validated in the bRIGHT cohort, and reported 1-year mortality rates after T-TEER ranging from 5% in early disease, to 33% in patients with advanced disease, with outcomes influenced by both baseline disease stage and right ventricular (RV) function. Patients with intermediate-stage disease derived the greatest survival benefit from T-TEER, whereas those with advanced disease experienced higher mortality, regardless of intervention.^20^

Comparatively, the TRISCEND-II trial reported a 30-day all-cause mortality rate of 3.5% and 1-year all-cause mortality rate of 11.6% after TTVR.^15^ Similarly, the TRI-SPA Replace registry reported a 30-day mortality of 2.2%, with TR grade ≤1+ in 94% of patients at 30 days.^21^ Registry data from the TriACT study reported a 30-day mortality of 8%, which can be compared to just 5.1% in a recent European registry analysis of patients treated with the EVOQUE system since system approval in Europe in October 2023.^22^^,^^23^

In summary, registry data indicate that 1-year mortality after T-TEER is approximately 15%–20%, with lower rates in patients achieving significant TR reduction. These mortality rates are comparable to those reported for TTVR in recent clinical trials and registries; however, direct comparisons are limited by differences in patient selection and baseline risk profiles.

### Procedural Complications

In the TRILUMINATE trial, freedom from major adverse events at 30 days was 98.9%, and at 1 year the rate of major bleeding was 5.2%, with permanent pacemaker implantation in 2.9% of patients. Device-related complications were infrequent: single-leaflet device attachment occurred in 7.0% of the attempted procedures, and there were no cases of device embolization or thrombosis. Tricuspid-valve surgery during follow-up was rare (1.8%).^9^^,^^12^ Registry data from the PASTE study, which included over 1000 high-risk patients, reported intraprocedural success in 85% and Tricuspid Valve Academic Research Consortium-defined clinical success at 1 year in 56%–87% depending on device generation and center experience.^10^ The overall major adverse event rate at 1 year was low, with most complications related to bleeding or vascular access, and device-related events remained rare.^10^

T-TEER is associated with infrequent periprocedural mortality (typically <2%) and a low complication rate (2%–8% in contemporary trials and registries). These rates are generally lower than those reported for TTVR, and although it achieves more complete TR reduction, TTVR is associated with higher rates of major bleeding, conduction disturbances and need for permanent pacemaker implantation than T-TEER.^15^^,^^16^^,^^24^ However, the complication rate following TTVR is steadily declining. In a real-world cohort, rates of severe bleeding complications were recently reported at 7.4%, which is compared to the higher 26.8 and 10.8% in the TRISCEND and TRISCEND-II trials, respectively.^15^^,^^22^^,^^25^ Similarly, the pacemaker rate, reported at 24.7% in the TRISCEND-II trial was 18.9% in pacemaker naïve patients in a real-world cohort at 30 days,^22^ with conduction disturbances predicted by preexisting conduction disturbances, but not annular dimensions on preprocedural imaging.^22^ Similarly, in the TRI-SPA Replace registry, 15.8% of the 48 patients required a permanent pacemaker after TTVR.^21^

The favorable safety profile of T-TEER is therefore a key consideration when weighing T-TEER against TTVR, and there should be an emphasis on individualizing the therapeutic strategy based on patient comorbidities, and procedural risk.

#### Transcatheter Tricuspid Valve Annuloplasty

The Cardioband system (Edwards Lifesciences) is the only transcatheter annuloplasty device with CE mark approval for the TV and is available in Europe. It is implanted via transfemoral venous access with anchors along the annulus and subsequent cinching to reduce annular size, mimicking surgical annuloplasty. Early studies, including TRI-REPAIR,^26^ TriBAND,^27^ and the Edwards Early Feasibility Study,^27^ report high technical success rates and reductions in annular diameter and TR; however, long-term durability and broader clinical impact remain under investigation, and owing to the broad indications for T-TEER, use of the Cardioband system has been limited.^24^^,^^28^

#### Heterotopic Transcatheter Therapies for Tricuspid Regurgitation

Heterotopic therapies are compelling where the tricuspid anatomy is unsuitable for either T-TEER or TTVR to mitigate regurgitant flow into the vena cava and directly address the systemic congestion contributing to HF symptoms, rather than treating TR. Early feasibility data across these systems report high procedural success rates and low periprocedural mortality. The TricValve system (P&F Products & Features, Vienna, Austria) is a self-expanding bicaval device implanted in the superior and inferior venae cavae that has CE Mark approval. Clinical trial data from multicenter registries and prospective studies demonstrate procedural success rates of 94%-96% for TricValve, with no procedural deaths and low device-related complications. At 1-year, clinical improvement was seen in 83%–95% of patients, with significant gains in New York Heart Association Functional Classification (NYHA) functional class, Kansas City Cardiomyopathy Questionnaire scores, and 6-minute walk distance. HF hospitalizations and diuretic requirements were reduced, and 1-year all-cause mortality ranged from 6.8 to 19.1%, with higher mortality in patients with elevated TRI-SCORE (see subsequently), reflecting advanced comorbidity.^29^, ^30^, ^31^ The Trillium system (Innoventric, Tel Aviv, Israel) is a cross-caval stent prosthesis, which was evaluated in a single-arm, multicenter study of 20 high-risk patients. Device implantation was successful in all cases. At 30 days, central venous pressure was significantly reduced, and there was a statistically significant improvement in NYHA functional class; however, whether these benefits translate into improved long-term outcomes remains to be established in randomized trials.^32^ Other heterotopic systems currently under development include the TRICENTO system (NVT GmbH, Hechingen, Germany), a bicaval covered stent that extends through the right atrium between the cavae, directing venous return toward the TV. Early follow-up in 21 patients demonstrated complete procedural success, no in-hospital mortality, and improved NYHA functional class.^24^ The Unica system (Innovalve; Innoventric, Vienna, Austria) is similarly a single-stent, dual-valve design for caval implantation. First in-human experience showed technical feasibility; however, trial data for this system is awaited.^33^ Collectively, these devices represent a distinct therapeutic category, offering a strategy for patients with severe TR and advanced comorbidities who are unsuitable for orthotopic repair or replacement.

A summary of the available transcatheter devices for TV intervention is in Table 1.

**Table 1:** Strategies for transcatheter tricuspid valve intervention.

Strategy	Approved devices∗	Indications	Contraindications	Trials and registries
T-TEER	TriClip (Abbott)/PASCAL (Edwards Lifesciences)	Symptomatic severe TR despite OMT; favorable anatomy.	Severe RV dysfunction; precapillary PH; coaptation gap >10 mm; severe leaflet tethering; adherent device leads	TRILUMINATE^9^^,^^12^^,^^13^; Tri.FR^14^; PASTE registry^10^
TTVR	EVOQUE (Edwards Lifesciences)	Symptomatic ≥ moderate TR despite OMT; anatomy unsuitable for T-TEER; adequate annular dimensions	Severe PH (SPAP >60–70 mmHg, PVR >4 WU); severe RV dysfunction; active endocarditis; life expectancy <12 mo; ICD leads	TRISCEND-II^15^; TRI-SPA Replace^21^; TriACT^22^^,^^23^
Annuloplasty	Cardioband (Edwards Lifesciences)	Symptomatic moderate–severe functional TR with significant annular dilatation, large coaptation gaps, and/or pronounced leaflet tethering, where T-TEER is less likely to be successful.	Severe leaflet restriction/calcification; severe RV dysfunction; large annular size beyond device range	TRI-REPAIR^26^; TriBAND^27^; Early Feasibility Study; Meta-analyses^28^^,^^29^
Heterotopic/CAVI	TricValve (P&F Products & Features)	Severe symptomatic TR not amenable to T-TEER/TTVR; symptomatic relief of elevated systemic venous pressures in advanced disease	Not suitable if caval anatomy incompatible; advanced comorbidity	TRICUS EURO, multicenter registries^30^, ^31^, ^32^

Abbreviations: CAVI, caval valve implantation; ICD, implanted cardiac defibrillator; OMT, optimal medical therapy; PH, pulmonary hypertension; PVR, pulmonary vascular resistance; RV, right ventricle; SPAP, systolic pulmonary arterial pressure; TR, tricuspid regurgitation; T-TEER, transcatheter edge-to-edge repair; TTVR, transcatheter tricuspid valve replacement; WU, Wood units.

∗ Devices with CE Mark approval.

## Preprocedural Assessment

Patients should be managed with OMT based on the underlying TR etiology. This includes medical therapy for cardiac failure, rhythm or rate control for atrial fibrillation, diuresis aiming to alleviate systemic venous congestion, and pulmonary vasodilators in the setting of precapillary pulmonary hypertension (PH). It is therefore essential that in addition to cardiac imagers and interventionalists, the heart team also comprises electrophysiologists and HF and PH specialists to advise on the medical optimization of patients being considered for tricuspid intervention and inform long-term treatment planning.

### Imaging

**Figure 1:**
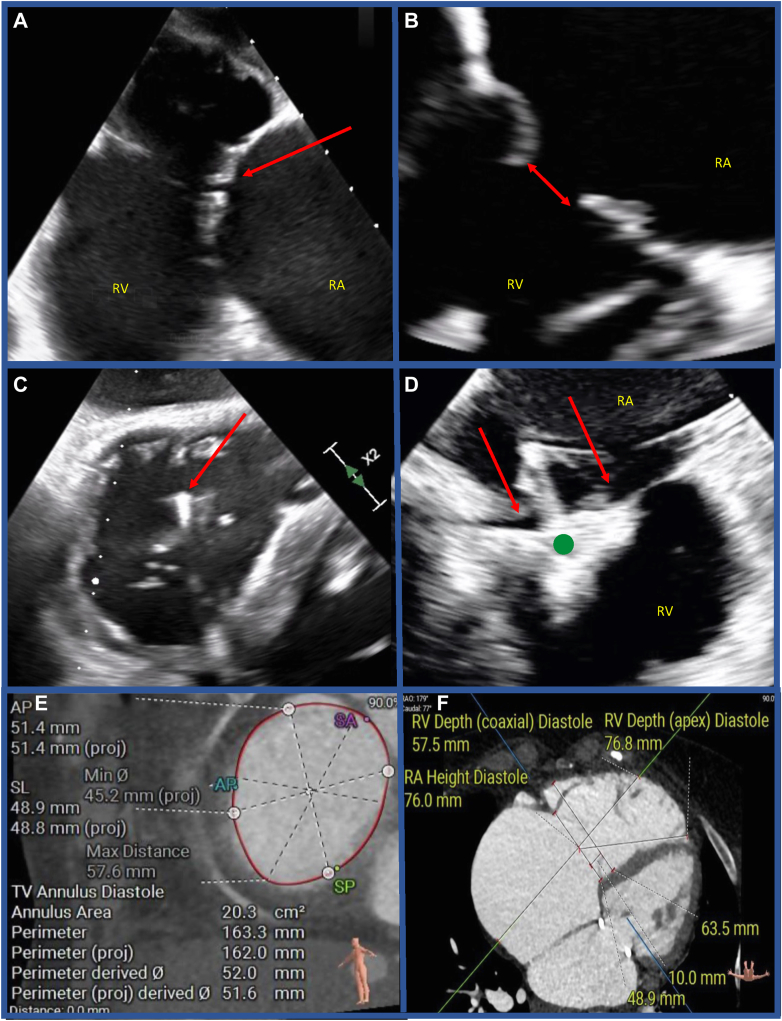
**Imaging of the tricuspid valve in TTVI. (a)** Transesophageal images showing adequate image quality with desirable anatomical features for T-TEER with good leaflet length, mobile leaflets, and a reasonable coaptation gap (red arrow). **(b)** Transesophageal echocardiographic images showing suboptimal anatomical features for tricuspid T-TEER, with a wide coaptation gap (red arrow). This patient was referred for TTVR. **(c)** Transesophageal echocardiographic images showing suboptimal image caused by artefact from a transvalvular pacing lead (red arrow). This patient underwent T-TEER supported by ICE. **(d)** Using ICE, good quality images of the clip (green dot) and valve leaflets (red arrows) were obtained during T-TEER, despite the presence of pacing leads, in the patient from Panel C. **(e)** CT annular dimensions in the diastolic phase, showing the anteroposterior and septolateral dimensions, as well as the annular perimeter. **(f)** CT 4-chamber view obtained during work-up for TTVI in a patient who was anatomically unsuitable for T-TEER. This section demonstrates the RV and RA heights, which in addition to annular dimensions comprise the CT screening parameters for TTVR Abbreviations: AP, anteroposterior; ICE, intracardiac echocardiography; RA, right atrium; RV, right ventricle; SA, septoanterior; SL, septo-lateral; SP, septoposterior; T-TEER, transcatheter tricuspid valve edge-to-edge repair; TV, tricuspid valve; TTVI, transcatheter TV intervention.

Multimodality imaging is the cornerstone for patient selection and procedural planning. Transthoracic and transesophageal echocardiography (TTE and TEE) are fundamental, not only for assessment of TR severity and anatomy, but also for the assessment of RV and right atrial size and function, as well as detecting untreated reversible left-sided disease.^34^^,^^35^ Three-dimensional (3D) echocardiography is essential for assessing valve anatomy and for guiding the positioning of the device. Baseline echocardiography should include TTE with TV imaging from in parasternal long and short, 4-chamber, and short-axis subcostal views. Basic TEE requires standard 2D midesophageal 4-chamber, RV inflow–outflow, and transgastric short-axis views. For T-TEER, intraprocedural guidance relies on biplane and 3D en face TEE views to identify leaflets, guide device trajectory, and confirm leaflet grasping. For TTVR, 3D TEE provides real-time confirmation of device positioning, anchoring, and paravalvular leak assessment, whereas computed tomography (CT) is essential for annular sizing and relation to surrounding structures (Figure 1).

In addition, cardiac CT is performed for cases considered for TTVR, offering excellent spatial resolution to assess tricuspid annular dimensions for device sizing, as well as the relationship of the annulus to surrounding structures including the right coronary artery, ostium of the coronary sinus, venae cava, and, when present, endocardial device leads. CT also allows planning for vascular access.^11^^,^^36^ Preprocedural imaging for transcatheter tricuspid interventions should ideally be performed in a euvolemic state. The right heart structures, including the right atrium, RV, and TV annulus are highly sensitive to fluctuations in intravascular volume. Volume overload can lead to chamber dilation, resulting in overestimation of the valve coaptation gap, as well as inaccurate annular measurements, which may contribute to valve oversizing in TTVR. As such, careful optimization of volume status is essential before imaging, with escalation of diuretic therapy when indicated. The patient’s weight should be recorded at the time of imaging and maintained within a narrow range up to the procedure to ensure consistency in anatomical dimensions.^11^

Cardiac magnetic resonance imaging is not routinely incorporated into the standard preprocedural workup for TTVI; however, it may provide complementary information in selected cases. Specifically, cardiac magnetic resonance imaging can offer accurate quantification of stroke volume and tricuspid regurgitant volume, as well as detailed myocardial tissue characterization, which may be valuable in guiding management in patients with suspected cardiomyopathy or complex biventricular pathology.^36^

Preprocedural imaging also serves to assess the anticipated quality of intraprocedural TEE, which is critical for procedural guidance, particularly during T-TEER. Adequate visualization of the TV leaflets is essential for successful leaflet grasping and device deployment. Identifying potential imaging limitations enables appropriate procedural planning and, if necessary, consideration of alternative imaging strategies or procedural approaches.^35^^,^^36^ In cases where TEE is contraindicated or gives suboptimal image quality, intracardiac echocardiography (ICE) is a viable adjunct in experienced centers.^11^^,^^35^ ICE offers several advantages, including the ability to perform procedures without the need for general anesthesia, potentially completely avoiding placement of a TEE probe and enabling the use of conscious sedation. However, ICE is associated with a learning curve, and also necessitates modifications to the standard procedural workflow.^37^ Although it is usually used in combination with TEE in complex cases, it is likely to play an increasing role in TTVI.^38^

### Right Heart Catheterization and Right Ventricular Function

Right heart catheterization (RHC) is routinely performed before TTVI to characterize the presence and severity of PH, with specific attention to mean pulmonary artery pressure (PAP) and pulmonary vascular resistance. These parameters are critical for procedural planning, risk stratification, and determining eligibility. Notably, major clinical trials of TTVR have excluded patients with systolic PAP (SPAP) > 60–70 mmHg or pulmonary vascular resistance >4 Wood units, resulting in limited outcome data for this high-risk population, who are often deemed ineligible for current transcatheter therapies.^9^^,^^11^^,^^25^

In select cases, particularly when the severity of TR is uncertain, right atrial V-wave measurement may offer additional diagnostic information. However, this marker is not universally reliable, particularly in patients with markedly dilated right atria, where pressure transmission is attenuated.^39^ Elevated mean right atrial pressure is however associated with lower procedural and higher mortality rates at 2 years after TTVI.^40^

As a low-pressure chamber, the RV is highly sensitive to changes in afterload. In chronic TR, the effective RV afterload is reduced due to regurgitant flow into the low-pressure RA. Following TTVI, particularly TTVR, regurgitation is substantially reduced or eliminated, leading to an acute increase in RV afterload.^34^ These hemodynamic changes were recently demonstrated in a cohort of 20 TTVR patients undergoing preprocedural and postprocedural RHC. As expected, TTVR led to an acute reduction in the right atrial and V-wave pressures, as well as RV and SPAP.^41^ Although the abrupt hemodynamic shifts after TTVR have raised concerns regarding the risk of acute postprocedural RV failure, especially in patients with preexisting PH, in this particular cohort, no instances of acute RV failure were observed; however, the baseline mean PAP was just 22.7 ± 5.1 mmHg. Although early clinical experience has not demonstrated a high incidence of acute RV failure, this may reflect the conservative inclusion criteria of the original trials,^15^^,^^42^ and in real-world cohorts, where instances of acute RV failure have been observed,^22^^,^^43^ this requires further hemodynamic and clinical investigation.

To better contextualize RV function in the setting of afterload, the concept of RV–pulmonary artery (RV–PA) coupling has emerged as an important prognostic tool.^44^ This parameter integrates contractility and afterload, offering insight into RV adaptation to afterload. Although traditionally estimated using echocardiographic metrics such as tricuspid annular plane systolic excursion to right ventricular systolic pressure, recent data suggest that the ratio of tricuspid annular plane systolic excursion to invasively measured SPAP provides superior prognostic accuracy.^45^ Combining TTE with RHC thus allows integrated assessment of RV–PA coupling. However, despite its incremental value, RV–PA coupling has not yet been universally adopted into routine clinical decision-making.^24^ Further studies and experience are needed to validate its prognostic thresholds and define its role in guiding patient selection and predicting RV recovery following TTVI.

## Patient Selection

The criteria for patient selection in the original T-TEER trials, such as TRILUMINATE, focused on patients with symptomatic TR, preserved RV function, and optimal anatomy for edge-to-edge repair.^9^^,^^46^ These parameters continue to define ideal candidates. However, as procedural experience has grown and device technology advanced, the scope of eligible patients has expanded to include those with more complex anatomical features and higher-risk clinical profiles.^47^ Currently, T-TEER is indicated for patients with >3+ TR who remain symptomatic despite OMT.^48^ Although many of these patients achieve meaningful improvements in symptoms and functional status, those with severe RV dysfunction or precapillary PH tend to have more limited prognostic benefit.^49^^,^^50^

Patient selection for TTVR is broadly defined by the criteria used in the TRISCEND-II trial, which required ongoing HF symptoms and moderate or greater TR despite OMT in patients without significant PH or among other significant comorbidities, severe left or RV dysfunction.^46^ General contraindications to TTVI are active endocarditis, life expectancy <12 months, and advanced PH or severe RV failure.^47^^,^^51^ Functional capacity should be objectively evaluated using tools such as the 6-min walk test, Kansas City Cardiomyopathy Questionnaire, and NYHA functional class.

However, although these criteria provide a foundation for patient selection and exclusion, they do not completely capture the nuanced decision-making required by the heart team. Multiple risk scores have been applied to TTVI populations, with the intent of summarizing procedural risk. This includes the TRI-SCORE, which was originally developed to predict in-hospital mortality after isolated TV surgery; however, recent studies have evaluated its utility in patients undergoing TTVI. Higher scores are associated with increased rates of in-hospital complications, 30-day mortality, and adverse outcomes.^17^ The TRIVALVE score was derived from the TRIVALVE registry specifically for TTVI and predicts 12-month mortality or rehospitalization, encompassing the high- and prohibitive-risk populations studied in the T-TEER and TTVR trials.^52^ Although these scores integrate elements of clinical and anatomical procedural risk, an in-depth understanding of the factors driving treatment decisions is still required to tailor TTVI strategies.

### Anatomical Considerations

Successful T-TEER depends on favorable TV anatomy for effective device placement. Optimally, this includes coaptation gaps ≤7 mm, a low tenting height, an anterior jet location, and an absence of leaflet tethering or endocardial device leads; however, more complex anatomies may still be treated successfully at experienced centers.^47^^,^^53^, ^54^, ^55^, ^56^, ^57^, ^58^, ^59^, ^60^, ^61^, ^62^ There is no absolute optimal tenting height, but rather this should be interpreted in the context of other anatomical factors. Structural abnormalities including leaflet thickening, tethering, or chordal density may limit procedural feasibility. Endocardial device leads, particularly if they are adherent to a valve leaflet, increase procedural complexity,^51^ and in general procedural success is dependent on adequate leaflet length, mobility, and accessibility.^53^ Access and delivery system maneuverability are influenced by the height, orientation, and offset of the inferior vena cava relative to the tricuspid annulus. Technical complexity may arise when the inferior vena cava orifice is steeply oriented and close to the tricuspid annulus, limiting atrial height for coaxial alignment of the delivery system,^63^ newer-generation delivery systems are generally capable of accommodating a range of anatomical variations. Additional technical complexity may arise from prominent chordae or subvalvular structures, which can impede catheter navigation, entangle the device, or increase the risk of iatrogenic injury. Such complications may worsen TR and prevent optimal procedural outcomes.^64^

To predict the likelihood of procedural success based on anatomy, the Gap, Location, Image, Density, En Face TV Morphology (GLIDE) score assesses the anatomical complexity for T-TEER, based on the preprocedural transesophageal echocardiographic assessment (Table 2)^65^ (Figure 1). Considering the low likelihood of procedural success for patients with GLIDE scores 4 or 5, these patients may be better treated with TTVR.

**Table 2:** Summary of the GLIDE score

Anatomical component	Straightforward	Complex
Septolateral coaptation gap	0-5 mm	≥6 mm
Location of predominant TR	Anteroseptal/central	Posteroseptal/anteroposterior/diffuse
Image quality	Good	Limited
Density of chordal structure	Minimal	Moderate/high
En face TR jet morphology	Linear/oval	Star-shaped

Scoring range: 0–5 points (sum of individual components).
• 0–1 points: High likelihood of procedural success (>90% TR reduction ≥2 grades and TR ≤ moderate). • 2–3 points: Intermediate likelihood (∼47–61% procedural success). • ≥4 points: Low likelihood of procedural success (∼14–17%).

Abbreviation: TR, tricuspid regurgitation.

Adapted from Gerçek et al, 202,4.^66^

By contrast, the specific anatomical considerations that determine eligibility and predict procedural success for TTVR are more focused on annular sizing. The tricuspid annular size and geometry determine device suitability. The annulus must be large enough to accommodate the device, but not so large as to preclude secure anchoring. Excessive annular dilation may increase the risk of device malposition or paravalvular leak.^36^ Extremes of annular size are a common reason for screen failure for TTVR, and with the intention of broadening eligibility, a range of implant sizes have been designed, for example, in the commercially available EVOQUE platform, in which a larger, 56-mm implant size has been recently introduced to accommodate larger anatomies.^11^ TTVR is less dependent on the anatomy of the leaflets and subvalvular apparatus; however, severe leaflet restriction, calcification, or abnormal subvalvular structures can complicate device seating and anchoring.^11^^,^^16^^,^^24^

To accommodate the wide anatomical variability of the TV and expand the applicability of TTVR, a range of prosthetic valve designs are currently under development, most of which remain in early feasibility or clinical trial phases. These devices use various anchoring mechanisms, including subannular fixation, radial force at the annular level, and leaflet grasping, sometimes supplemented with additional stabilizing elements positioned in the RV outflow tract or right atrium to enhance device security. Delivery systems are typically designed for either transfemoral or trans-jugular access, with the latter offering a more direct and coaxial trajectory to the tricuspid annulus, potentially facilitating more straightforward deployment.^11^

## Patient Preconditioning

### Diuresis

From a procedural standpoint, adequate diuresis can reduce RV volume and annular dimensions, reducing the coaptation gap, and thereby improving leaflet approximation during T-TEER. For TTVR, maintaining euvolemia is particularly important, as the tricuspid annulus, right atrium, and RV are highly sensitive to shifts in intravascular volume and can dilate significantly with volume overload.^11^^,^^24^ Although annular dimensions are reassessed intraprocedurally using multiplanar TEE, aligning the patient’s volume status from the outset enhances procedural precision and minimizes the risk of sizing discrepancies.^35^

From a clinical standpoint, preprocedural optimization can improve procedural safety and recovery. TR causes RV volume loading, contributing to ventricular impairment and systemic venous congestion, with edema, pleural effusions, renal impairment, and gastrointestinal tract and hepatic congestion, causing hepatic impairment, ascites, abdominal pain, and anorexia.^66^^,^^67^ Optimization of preload and systemic congestion is essential to improve end-organ function and procedural recovery. Aggressive preprocedural decongestion with diuretic therapy can improve end-organ function, reduce edema, improve mobility, and support recovery.^47^ However, diuretic resistance is common in this population, particularly in the context of long-standing HF. Hospitalization for preprocedural optimization is therefore often required. Advanced strategies such as intravenous diuretics (including continuous loop diuretic infusions), inotropic support, or ultrafiltration may be necessary in selected patients.^47^^,^^68^

Organ function should be closely monitored and optimized before intervention. Congestive hepatopathy and cardiorenal syndrome, common in advanced TR, are associated with increased procedural risk. Where practicable, exposure to hepatotoxic or nephrotoxic agents should also be reduced during this period.^68^

### Pulmonary Hypertension

The management of coexisting PH is also a key determinant of procedural safety and long-term outcomes. Preprocedural optimization includes careful assessment of volume status, RV function, and RV–PA coupling, with the goal of minimizing the risk of acute RV decompensation, particularly relevant in TTVR.^44^^,^^69^ Because echocardiographic estimates of pulmonary pressures may be unreliable in the setting of severe TR, invasive hemodynamic assessment is recommended to accurately characterize pulmonary pressures and RV afterload.^11^^,^^45^ For patients with precapillary PH, pulmonary vasodilator therapy should be considered and optimized in consultation with PH specialists. Targeted pulmonary vasodilator therapies are not routinely indicated in the absence of a precapillary component. According to current recommendations, the management of isolated postcapillary PH should instead focus on optimizing left-sided filling pressures, treating underlying cardiac causes and implementing rhythm or rate control strategies, particularly in the setting of atrial fibrillation. In rare cases with reversible precapillary PH, selective vasodilator therapy may be considered; however, this remains an exception rather than the rule for most patients undergoing TTVI.^24^

In select cases, repeat RHC may be warranted to reassess pulmonary pressures after medical therapy and confirm suitability for intervention. Aggressive volume optimization with diuresis can also lead to a reduction in pulmonary pressures and improved RV function.^24^^,^^69^ The presence of severe PH, typically defined as systolic pulmonary arterial pressure >70 mmHg, or severely impaired RV–PA coupling identifies patients at elevated risk for RV failure following TTVR. As such, these patients are frequently excluded from early feasibility trials and may not be appropriate candidates for current TTVR options.^11^^,^^70^ In contrast, T-TEER may still be considered in selected patients with severe PH and, where procedural success and symptomatic improvement remain achievable.

The periprocedural management of anticoagulation is important in this population due to a high background rate of atrial fibrillation and elevated bleeding risk. This is discussed subsequently.

## Procedure-Specific Considerations

In addition to anatomical and clinical characteristics, similarly to mitral TEER, procedural success with T-TEER may be dependent on the experience and expertise of the implanting center and heart team.^71^^,^^72^ This includes both the interventionalist and echocardiographer, who must collaborate to develop a tailored, case-by-case strategy based on the patient’s unique anatomy. High-quality intraprocedural imaging guidance is essential for optimal outcomes.^35^ Although low-volume centers may achieve favorable results in patients with favorable anatomy, such as those with GLIDE score 0 or 1, more complex cases are likely to benefit from treatment at high-volume centers with extensive and long-standing experience and established heart teams.

Beyond the immediate procedural considerations, the experience and continuity of care provided by the heart team play a central role in longer-term success. Although technical success is influenced by preprocedural optimization and operator skill, postprocedural management is increasingly recognized as critical, particularly given the impact of TTVI on RV function. Following valve repair or replacement, RV function is often acutely impaired due to the sudden increase in afterload. Over time, however, favorable RV remodeling may occur, characterized by a reduction in RV volumes and gradual recovery of systolic function.^73^ During this period, close monitoring and medical management are essential. Optimization of diuretic therapy, volume status, renal and hepatic function, and neurohormonal blockade can support this remodeling process and mitigate the risk of recurrent symptoms or worsening of TR. Importantly, abrupt cessation of diuretics, particularly in patients previously requiring high doses, may lead to recurrent RV dilatation, annular distortion, or even leaflet detachment. Thus, structured, long-term follow-up by an experienced multidisciplinary team is essential to consolidate procedural gains and sustain clinical improvement.^55^

### Imaging at Follow-up

Currently, TTE is the recommended imaging strategy for assessment of valve function after TTVI, including residual TR and valve gradient. For TTVR, however, postprocedural cardiac CT is performed routinely at some centers. CT has superior spatial resolution, and greater sensitivity to detect valve leaflet thrombosis or hypoattenuated leaflet thickening (HALT) compared to TTE. Although the natural history and clinical significance of HALT in TTVR is not yet widely documented,^74^ there is extensive evidence in transcatheter aortic valve replacement showing a high incidence and poorer clinical outcomes with HALT.^75^, ^76^, ^77^, ^78^, ^79^, ^80^ Due to lower pressure gradients and flow, as well as greater surface area, HALT could be expected to be prevalent on the TV, and as such, in these early stages of our experience, routine postprocedural CT is advised in addition to TTE to establish the burden of thrombosis and HALT, which may guide changes in anticoagulation strategies.

### Preexisting Endocardial Device Leads

Endocardial device leads are common in patients referred for TTVI and introduce additional procedural complexity in both T-TEER and TTVR.^46^ Given the high prevalence of conduction system disease in the elderly population, a substantial proportion of patients present with preexisting endocardial pacing or defibrillator systems, which may themselves contribute to the development or worsening of TR via leaflet tethering or entrapment.^51^

In the context of TTVR, the presence of endocardial leads raises specific concerns. TTVR devices exert radial force at the annular level, increasing the risk of mechanical interaction with preexisting leads. This is particularly relevant in patients with pacing dependency, where the risk of lead impingement or fracture may result in lead dysfunction, posing significant clinical consequences.

Transvalvular leads may also preclude adequate positioning and anchoring of the TV prosthesis, and although lead extraction can be considered, this could impose technical difficulties, and it is associated with a significant complication rate. These procedural and anatomical risks require careful preprocedural planning, ideally with multidisciplinary input, including electrophysiology consultation to assess pacing dependency, and lead positioning, and document lead impedance, so it may be closely monitored postprocedurally. Discussion regarding lifetime planning of both the valve and pacing devices should also be undertaken as part of the initial procedural planning. Due to the uncertain long-term safety profile, TTVR is currently not supported in patients with endocardial implantable cardioverter-defibrillator leads.^11^

The long-term risk of endocarditis is unknown, including in patients with device leads, which may pose an additional risk. As such, life-long endocarditis prophylaxis has been recommended. Considering the frailty and comorbidity profile of patients in TTVI cohorts, conservative management with lifelong antibiotic suppression therapy is recommended as the first line for cases of endocarditis.^11^

### Postprocedural Conduction Disturbances and Pacing Strategies

TTVR may result in new conduction disturbances, including complete heart block, particularly in patients with baseline conduction disease such as left bundle branch block. During valve implantation, mechanical compression of the right bundle branch may precipitate atrioventricular block. As such, implanting teams should be prepared for temporary pacing, with backup systems in place for intraprocedural management.

For patients requiring permanent pacing after TTVR, traditional trans-tricuspid endocardial lead placement is not favored due to the risk of prosthesis dysfunction. Alternative pacing strategies must be considered, including coronary sinus lead placement for left ventricular pacing, epicardial systems, which are more invasive, requiring surgical access, or leadless pacemaker technologies, which offer promising alternatives without continuously traversing the TV. This reinforces the importance of early consultation and collaboration with electrophysiologists within the heart team to guide preprocedural planning and postprocedural device management. The expanding use of TTVR is also likely to drive innovation in leadless and extracardiac pacing devices to overcome the challenges associated with pacing systems in TTVI.

### Bleeding, Thrombosis, and Anticoagulation

The periprocedural anticoagulation strategy requires careful individualized consideration. Severe TR is associated with an increased bleeding risk, with a background of rate of up to 15% annualized major bleeding, owing, among other factors, to elevated systemic venous pressures, renal and hepatic dysfunction, and anticoagulation.^4^

In the TRISCEND study, of the 56 patients who underwent TTVR, 19.6% had a history of gastrointestinal bleeding at baseline. After valve implantation, 26.8% (15 patients) had a major or severe bleeding event,^81^ with 9 requiring blood transfusion. All were taking anticoagulation or an antiplatelet agent before the procedure, 73% a vitamin K antagonist (VKA), however, with an average preprocedural INR of just 1.6.^25^ Similarly, in the TRILUMINATE trial by contrast, just 4.7% of the 175 patients in the intervention cohort experienced major bleeding at 30 days and 5.2% by 1 year.^9^ At 2-year follow-up of this trial, this had risen to 11.9%.^8^ Although a proportion of this will be driven by periprocedural bleeding events, for example, from the access site or the esophagus from TEE imaging, the bleeding risk in this population is heightened and requires careful attention.

The majority of patients are already anticoagulated before the procedure, most commonly for atrial fibrillation. Although there is currently no standardized anticoagulation regimen for TTVR, anticoagulation is recommended due to the risk of valve thrombosis, particularly in the low-flow, low-pressure environment of the right heart, where blood stasis may promote valve leaflet thrombus formation.^11^ Although VKAs were initially favored, the use of non-VKA oral anticoagulants (direct oral anticoagulants) has become increasingly common in routine clinical practice, with a trend toward maintaining the same postprocedurally as before their procedure, with a change to VKAs only in the context of significant renal impairment, or where valve thrombosis is observed early in the postoperative course. Importantly, patients who cannot tolerate anticoagulation are not currently considered candidates for TTVR. Patients with unacceptable bleeding risk for long-term anticoagulation can, however, be considered for T-TEER, which, if efficacious, offers the possibility of reducing systemic venous congestion and therefore bleeding risk.

The long-term thrombotic risk profile following TTVR remains incompletely defined. Although HALT is a well-recognized phenomenon after transcatheter aortic valve replacement,^76^ reports of HALT following TTVR are currently limited.^74^ This may reflect the relatively early phase of device adoption and imaging follow-up, and HALT may be more prevalent than currently appreciated.^82^ In line with this, routine 30-day cardiac CT can be considered as part of routine follow-up, to monitor for subclinical valve thrombosis, especially until the prevalence and risk factors for HALT and anticoagulation strategies are more completely defined.

Finally, the concomitant use of antiplatelet agents warrants careful consideration, as these further naturally increase bleeding risk. When antiplatelet therapy is indicated, such as in patients with recent percutaneous coronary intervention, antiplatelets should be continued; however, conscious efforts should be made to discontinue therapy as soon as clinically appropriate.

## Summary and Recommendations

The decision between T-TEER and TTVR is nuanced and cannot be based solely on anatomical considerations, clinical risk scores, or a single numeric risk score threshold. Instead, a comprehensive, multidisciplinary assessment is required, beginning with evaluation by experienced clinicians from the heart team, with particular attention to comorbidities, medications, functional status, and specific limitations or requirements that will guide device selection.

Initial assessment should be accompanied by TTE, focusing on right and left ventricular function, atrial size, TR severity, as well as coexisting valve pathology. Laboratory studies should include renal and hepatic function tests and a full blood count to identify preexisting anemia or organ dysfunction, which may impact procedural risk, particularly bleeding and tolerance of anticoagulation. An electrocardiogram should be performed to assess for baseline conduction disease, and risk for subsequent pacing requirement. For patients with preexisting pacemakers, information should be obtained regarding the type of device, lead configuration, battery life, and pacing dependency. Early consultation with an electrophysiologist is recommended to inform procedural planning, particularly if device–lead interaction is anticipated, the patient is pacing dependent or there is a considerable expectation for permanent pacing requirement after TTVI. For patients with implanted defibrillators, TTVR is not currently recommended; however, T-TEER can be performed, providing this is anatomically feasible in the presence of the endocardial leads.

At initial assessment, medical therapy should be reviewed and optimized. This includes HF therapy, diuresis to maintain euvolemia, rhythm and/or rate control for atrial fibrillation, as well as cardiac resynchronization therapy and pulmonary vasodilators if indicated.

Patients with limited life expectancy, such as those with end-stage malignancy or advanced comorbid conditions, are not considered candidates for T-TEER or TTVR. Although previously referred for medical management, heterotopic (caval) valve implantations, therefore, have become an option for patients for whom T-TEER and TTVR are not suitable. Similarly, patients with a contraindication to anticoagulation are not eligible for TTVR, and anticoagulation planning must also be considered for those undergoing T-TEER.

Patients who are considered candidates for TTVI should be referred for TEE, ideally performed in a medically optimized, euvolemic state to ensure accurate anatomical evaluation of TV anatomy and TR severity to determine candidacy and procedural strategy for T-TEER. Attention should also be given to contraindications or complications for TEE, and image quality. Where TEE is contraindicated, or there are significant technical limitations impairing image quality, select cases should be considered for ICE at an experienced center. Before TTVI, right heart catheterization is recommended to assess for severe PH, which may contraindicate TTVI, and to identify precapillary PH, which may require specific pulmonary vasodilator therapy for optimization before consideration for intervention.

Individual cases should then be reviewed in a dedicated heart team meeting. Patients with favorable anatomy may proceed to T-TEER. If anatomical or technical concerns arise, consultation with a more experienced heart valve center should be supported, where the patient may also be considered for high-risk T-TEER, or evaluated for TTVR. In such cases, contrast-enhanced venous-phase cardiac CT is required to assess annular dimensions, leaflet anatomy, and relationships to adjacent structures. At present, the EVOQUE valve is the only commercially approved TTVR device in the United States and Europe; however, other systems are available at select centers under clinical trial.

T-TEER remains the most commonly used transcatheter treatment for TR worldwide. This is largely due to the wide availability of TEER devices, which were adapted from mitral valve technology and are supported by extensive clinical registry data. T-TEER is, therefore, typically the first-line transcatheter approach in high-risk patients with suitable anatomy, particularly in regions with established operator expertise and device access.

**Figure 2:**
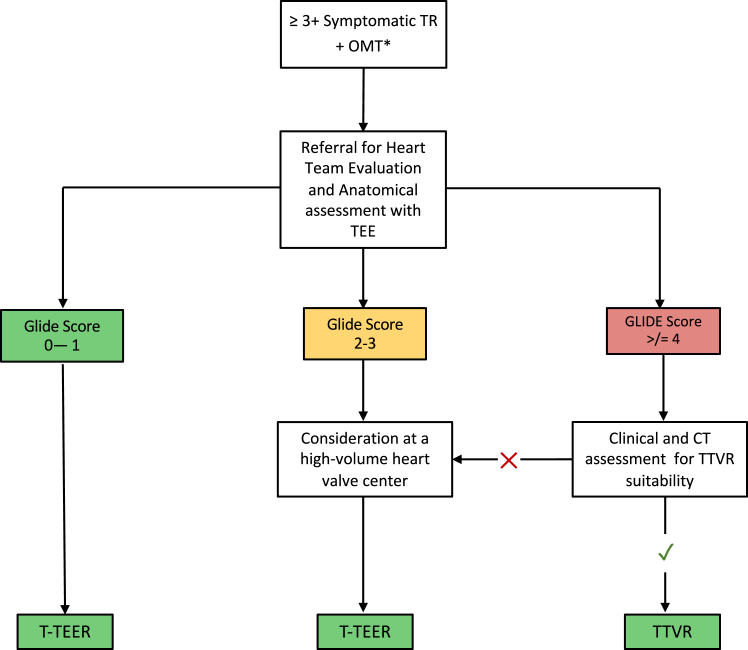
**Recommended decision algorithm for intervention for severe tricuspid regurgitation** Abbreviations: CT, computed tomography; OMT, optimal medical therapy; TEE, transesophageal echocardiogram; TR, tricuspid regurgitation; T-TEER, transcatheter tricuspid edge-to-edge repair; TTVR, transcatheter tricuspid valve replacement. ∗Optimal medical therapy, as per by guideline recommendations for valve disease related cardiac failure.

Ultimately, although anatomical and clinical risk factors are essential for procedural planning, the final treatment strategy must incorporate broader considerations, including lifetime device planning, both of the TV and pacing devices, bleeding risk and long-term anticoagulation strategy, operator and institutional experience, and health care infrastructure, device, and clinical trial availability and procedural cost. A suggested algorithm for procedural decision-making is presented in Figure 2.

## Future Directions

Significant progress has been made in the development of TTVI technologies, with an ever-increasing number of therapeutic options for a range of indications and anatomies. Despite this progress, cost and limited device availability remain substantial barriers to access in many centers. However, as more devices complete clinical trials and gain regulatory approval, it is anticipated that increased market competition will reduce costs. Meanwhile, it is important that clinicians and multidisciplinary heart teams remain abreast of the latest developments to ensure that patients are offered the most appropriate and up-to-date treatment options.

Leadless pacing options that do not interfere with transcatheter TV prostheses (and vice versa) are currently limited. This represents a significant gap and underscores the need for further innovation in device design and integration.

Uncertainty also persists regarding the optimal anticoagulation strategy following TTVR. Further studies are required to elucidate the incidence and natural history of HALT in this context, as well as to establish a therapeutic balance between thrombotic and bleeding risks.

Another area warranting further investigation is the hemodynamic threshold beyond which the RV decompensates due to excessive afterload. Specifically, the optimal threshold for RV–PA coupling that defines procedural safety, particularly in the context of TTVR, has yet to be determined. It is likely that patients included in clinical trials represented a conservative subset, and forthcoming registry data will be central to refining these parameters.

Imaging challenges also persist, especially in patients with suboptimal acoustic windows. The growing adoption of ICE may help to overcome some of these limitations and facilitate procedural planning and guidance; however, a significant learning curve will be required for this technology to become widely implemented.

Finally, with the expanding availability and applicability of transcatheter TV technologies, the feasibility of multivalve transcatheter interventions warrants consideration. Given the high prevalence of multivalvular disease, reported to be approximately 10% across all patients diagnosed with valvular heart disease, and increasing with the aging population, integrated percutaneous treatment strategies will become increasingly relevant.^83^

## Conclusions

Transcatheter therapies have transformed the management of TR, offering effective alternatives for patients at high surgical risk. T-TEER remains the most widely used approach due to its safety profile and the broad experience and availability, whereas TTVR offers more complete TR elimination, including in cases where the anatomy for T-TEER is unfavorable. Careful patient selection remains essential, integrating anatomical, clinical, and hemodynamic factors. Multidisciplinary heart team assessment, supported by multimodality imaging is critical to guide decision-making. As the field evolves, innovation in new devices, leadless pacing, cardiac and intracardiac imaging, and multivalve interventions will continue expand treatment options. Ongoing clinical trials and registry data will be the key to refining procedural indications and improving patient outcomes.

## Funding

This article was developed as part of the SHJ Mentorship Program, which is made possible by grant support from Edwards Lifesciences.

## Disclosure Statement

Lukas Stolz received speaker honoraria from 10.13039/100006520Edwards Lifesciences. Professor Fabien Praz has received travel expenses from 10.13039/100011949Abbott Vascular, 10.13039/100006520Edwards Life Sciences, Medira, 10.13039/100015696Siemens Healthineers, and InQBB Medical Technologies, and a research grant to the institution from 10.13039/100011949Abbott Vascular. Jörg Hausleiter reports research grant support and speaker honoraria from 10.13039/100006520Edwards Lifesciences.

The other authors had no conflicts to declare.
